# Utilization Strategies of Two Environment Phenotypes in Genomic Prediction

**DOI:** 10.3390/genes13050722

**Published:** 2022-04-20

**Authors:** Qing Lin, Jinyan Teng, Xiaodian Cai, Jiaqi Li, Zhe Zhang

**Affiliations:** Guangdong Provincial Key Lab of Agro-Animal Genomics and Molecular Breeding, Lingnan Guangdong Laboratory of Modern Agriculture, National Engineering Research Centre for Breeding Swine Industry, College of Animal Science, South China Agricultural University, Guangzhou 510642, China; qing_lin1996@126.com (Q.L.); kingyan312@live.cn (J.T.); xiaodian_cai@163.com (X.C.); jqli@scau.edu.cn (J.L.)

**Keywords:** genomic prediction, multiple environment phenotypes, genomic feature best linear unbiased prediction, multi-trait genomic best linear unbiased prediction, genome-wide association study, rice

## Abstract

Multiple environment phenotypes may be utilized to implement genomic prediction in plant breeding, while it is unclear about optimal utilization strategies according to its different availability. It is necessary to assess the utilization strategies of genomic prediction models based on different availability of multiple environment phenotypes. Here, we compared the prediction accuracy of three genomic prediction models (genomic prediction model (genomic best linear unbiased prediction (GBLUP), genomic best linear unbiased prediction (GFBLUP), and multi-trait genomic best linear unbiased prediction (mtGBLUP)) which leveraged diverse information from multiple environment phenotypes using a rice dataset containing 19 agronomic traits in two disparate seasons. We found that the prediction accuracy of genomic prediction models considering multiple environment phenotypes (GFBLUP and mtGBLUP) was better than the classical genomic prediction model (GBLUP model). The deviation of prediction accuracy of between GBLUP and mtGBLUP or GFBLUP was associated with the phenotypic correlation. In summary, the genomic prediction models considering multiple environment phenotypes (GFBLUP and mtGBLUP) demonstrated better prediction accuracy. In addition, we could utilize different genomic prediction strategies according to different availability of multiple environment phenotypes.

## 1. Introduction

Although the traditional breeding program had achieved notable genetic improvements in most livestock and crop species, further efforts are still needed to meet the increasing food needs in future decades. Genomic prediction (GP) [1] is one of the crucial methods that can efficiently improve genetic gain of agronomic traits by using genome-wide genetic markers. After the first elucidate of GP method (2001), VanRaden [2] proposed a common approach, the genomic best linear unbiased prediction (GBLUP), which was based on the genomic relationship matrix (GRM) to estimate the genomic estimated breeding values (GEBVs). Comparing with the traditional pedigree-based relationship matrix, the GRM could more effectively capture the effect of markers which link with the quantitative trait locus (QTL). Many simulative and empirical studies have shown that the GRM is superior to the pedigree-based relationship matrix for genetic evaluation in animal [3], plant [4], and aquaculture [5]. Based on the previous studies, a series of genomic prediction models were proposed or modified aiming to improve the prediction accuracy, such as single-step genomic best linear unbiased prediction (ssGBLUP) [6] and the others [7,8]. These models had various assumptions about the statistical distribution of the marker effects. However, they only took the statistical distribution into consideration and neglected the biologically complex genetic architecture.

Recently, numerous genomic prediction studies have shown that the genetic architecture of complex quantitative traits might affect the accuracy of genomic prediction [9,10]. Then, aiming to improve the prediction accuracy, a number of innovative genomic prediction models were proposed to incorporate the information of genetic architecture. Zhang [11] proposed a novel strategy, BLUP|GA, which could integrate the prior knowledge derived from a genome-wide association study (GWAS) of public databases into the genomic prediction model. In addition, Spindel [12] suggested that incorporating the genetic architecture and population structure explained by GWAS into the genomic prediction model might become an effective strategy for improving the prediction accuracy. Besides, a meaningful approach proposed, called “de novo GWAS”, which aimed to select the markers from GWAS, results in the training population and using these markers as fixed effects in the GBLUP model [13]. Furthermore, Edwards [14] developed a genomic feature best linear unbiased prediction (GFBLUP) that distinguished the weight of the individual genetic relationships by the genomic features, such as gene ontology. Moreover, the gene annotation or the estimated marker effects could be utilized to weight the genomic relationships to improve the reliability of GP [15,16]. In general, GP, considering the genetic architecture, could generally improve the prediction accuracy.

Due to the particularity of the plant breeding, one trait could be measured in multiple environments, and it could be utilized in GP to improve the prediction accuracy. There were several strategies to make use of the multiple environment phenotypes in GP. The first strategy was to take the non-additive effect into account in the GP model. Several studies showed that combining genotype by environment interaction (G × E) into the GP model could improve the GP performance [17,18]. The second strategy was that the same traits measured in different environments were served as a distinguishing and correlated trait which could be utilized in multi-trait genomic prediction model. Several simulation and empirical studies showed that the multi-trait genomic prediction model had superior prediction accuracy over the single-trait genomic prediction model for the low heritability traits [19,20]. However, the usage of multi-trait GP model was limited by data availability, because multiple-environment phenotypes required more cost of breeding and time. Therefore, it is important to use diverse GP models according to the different availability of the multiple environment phenotypes in the plant breeding program.

In this study, we used a set of elite rice breeding lines from the International Rice Research Institute (IRRI) irrigated rice breeding program to implement GP. The objective of this study was: (a) to compare the prediction accuracy of different GP models, and (b) to assess different utilization strategies in different availability of multiple environment phenotypes in GP.

## 2. Materials and Methods

### 2.1. Rice Population and Phenotype

The phenotypic (dry and wet season) and genotypic dataset, including 342 rice elite breeding lines, were downloaded from the Rice Diversity Panel [12,21] (http://www.ricediversity.org/data/index.cfm, accessed on 30 December 2019). This dataset was selected for phenotyping and genotyping from the International Rice Research Institute (IRRI) irrigated rice breeding program. Furthermore, a total of 19 agronomic, grain, and yield-related traits, consisting of plant height (PH), flowering date (FLW), culm length (culmL), number of effective tiller or panicle per plant (PN), panicle length (PL), flag leaf length (FlgLL), flag leaf width (FlgLW), flag leaf area (FlgLA), number of spikelet per panicle (SPn), number of filled grain per plant (FGP), grain length (GrL), grain width (GrW), grain length-breadth ration (LBR), lodging score (LG), peduncle length (PedL), panicle exertion rate (Exs), 1000 grain weight (1000 GW), yield per plant (YPP), and grain yield per plot (YLD), were recorded in both the dry (DS) and wet season (WS) for the years 2009–2012. The details of this rice breeding population can be viewed in ref. [12].

### 2.2. Genotype

Genotypic data of this rice breeding population contained 108,024 single nucleotide polymorphisms (SNPs). SNPs were filtered using the following criteria: (1) SNPs call rates of <0.9; (2) lines call rates of <0.9 and minor allele frequencies (MAF) <0.01 step by step using PLINK (version 1.90) [22]. After that, the missing maker genotypes were phased and imputed simultaneously using BEAGLE (version 5.1) [23] with the default parameters setting. A total of 74,251 SNPs and 342 lines met these criteria and were retained. Furthermore, the lines with missing phenotypes for both seasons were eliminated. Finally, 323 lines and 74,251 SNPs were used for further analyses.

### 2.3. Genome-Wide Association Study (GWAS)

GWAS was implemented in R statistical platform using GEMMA (version 0.98.1) [24]. The standard linear mixed model was performed, which was expressed as:

(1) y=Wα+Xβ+Zu+ε,

where y is an n-vector of the phenotypes for n lines; α is a vector of fixed effect, including the $1_{n}$ vector; β is an n-vector of marker genotypes; u is the random effect and it is set as $u\sim N\left(0,G\sigma_{u}^{2}\right)$, where G is the genomic relationship matrix; W, X, and Z are incidence matrices for α, β, and u; ε is the random residuals and consistent with $\varepsilon \sim N\left(0,I\sigma_{\varepsilon }^{2}\right)$, where I is an identify matrix. The genomic relationship matrix G was also calculated using GEMMA (parameter k = 1).

### 2.4. Genomic Prediction Models

Aiming to evaluate the prediction accuracy using different multiple environment phenotypes, three genomic prediction models were mentioned later. GBLUP, GFBLUP, and multi-trait genomic best linear unbiased prediction (mtGBLUP) models were expressed as Equations (2)–(4), respectively.

(2) $$y=1_{n}\mu +g+e,$$

(3) $$y=1_{n}\mu +g+g_{f}+e,$$

(4) $$\left[\begin{array}{c} y_{d}\\ y_{w} \end{array}\right]=\left[\begin{array}{c} \mu_{d}\\ \mu_{w} \end{array}\right]+\left[\begin{array}{c} \begin{array}{cc} Z_{d} & 0 \end{array}\\ \begin{array}{cc} 0 & Z_{w} \end{array} \end{array}\right]\left[\begin{array}{c} g_{d}\\ g_{w} \end{array}\right]+\left[\begin{array}{c} e_{d}\\ e_{w} \end{array}\right],$$

where y was the vector of the phenotypic values for each trait. μ was the overall mean of phenotypic values. $g\sim N\left(0,K_{1}\sigma_{g1}^{2}\right)$ was the vector of additive genetic effect. $g_{f}\sim N\left(0,K_{2}\sigma_{g2}^{2}\right)$ was the vector of specific additive genetic effect. The $\sigma_{g1}^{2}$ and $\sigma_{g1}^{2}$ were the additive genetic variance for all markers and for specific selected markers, respectively. $e\sim N\left(0,I\sigma_{e}^{2}\right)$ was a vector of residual effect where I and $\sigma_{e}^{2}$ were an identity matrix and the residual variance separately. The details of genomic relationship matrices $K_{1}$ and $K_{2}$ were described in the following section.

In Equation (4), Z was the design matrix for additive genetic effect. $\left[\begin{array}{c} g_{d}\\ g_{w} \end{array}\right]\sim N\left(0,V_{g}⊗K_{1}\right)$ was the additive genetic effect, and $V_{g}=\left[\begin{array}{c} \begin{array}{cc} \sigma_{g_{d}}^{2} & \sigma_{g_{\mathrm{dw}}} \end{array}\\ \begin{array}{cc} \sigma_{g_{\mathrm{wd}}} & \sigma_{g_{w}}^{2} \end{array} \end{array}\right]$ was the variance–covariance of additive genetic effect. $\left[\begin{array}{c} e_{d}\\ e_{w} \end{array}\right]\sim N\left(0,V_{e}⊗I\right)$ was the residual effect and $V_{e}=\left[\begin{array}{c} \begin{array}{cc} \sigma_{e_{d}}^{2} & \sigma_{e_{\mathrm{dw}}} \end{array}\\ \begin{array}{cc} \sigma_{e_{\mathrm{wd}}} & \sigma_{e_{w}}^{2} \end{array} \end{array}\right]$ was the variance–covariance of residual effect. The subscript of d and w represented dry season and wet season, respectively. Besides, the genetic parameters were estimated by Equation (4).

### 2.5. Genomic Relationship Matrices

The genomic relationship matrix K was constructed in line with the method originated from VanRaden [2] for all genomic prediction models, which was defined as

(5) $$G=\frac{ZZ^{T}}{2\sum_{i=1}^{m}p_{i}\left(1-p_{i}\right)},$$

where the matrix Z was the minor allele frequency (MAF) adjusted genotype matrix with the elements (0–2$p_{i}$), (0–2$p_{i}$), and (0–2$p_{i}$) for homozygote, heterozygote, and other homozygote, respectively. The m represented the number of markers. For the locus i, $p_{i}$ was the MAF of the ith SNP and $1-p_{i}$ was the other.

(1) All of the markers are utilized to construct the genomic relationship matrix G and applied in Equations (2)–(4) as $K_{1}$.
(2) In addition, with the purpose of comparing prediction accuracy for different availability of multiple environment information, the GWAS summary statistics from the other environment were leveraged to sort the markers from minimum to maximum by the column of *p*-values. After that, 60 genomic relationship matrices $G_{F}$ were constructed with the top percentage markers (from the top 1% to top 60% with step of 1%), which was implemented to Equation (3) as $K_{2}$.
(3) Furthermore, in order to confirm the role of multiple environment information, the percentage of markers, in the same as $G_{F}$, selected randomly was leveraged to construct the genomic relationship matrices $G_{R}$. the relationship matrices $G_{F}$ and $G_{R}$ were applied in Equation (2) as $K_{1}$.

Using average information restricted maximum likelihood (AI-REML) [25] via the regress package [26] in R statistical platform [27], and variance components of these model were estimated to be utilized in the cross-validations.

### 2.6. Predictive Ability Evaluation

**Figure 1:**
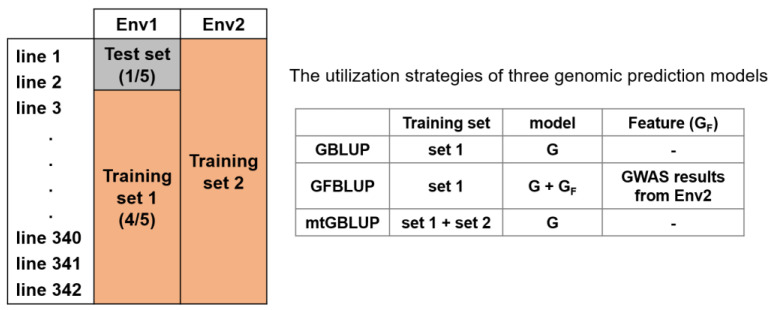
A scheme of cross validation using multiple environment phenotypes for genomic prediction. mtGBLUP model directly utilizes the Env2 phenotypes, while GFBLUP model only leverages the Env2 information (GWAS summary statistics). Env1/Env2 = Dry season or Wet season, GWAS = Genome-wide association study. G = Genomic relationship matrix using all markers, GF = Genomic feature relationship matrix using specific selected markers.

A five-fold random cross-validation (CV) was utilized to assess prediction accuracy for all genomic prediction models, shown in Figure 1. For each CV test, the rice lines were randomly divided into five groups on average. Four of five-fold were treated as a training set, and the other one served as a test set. In the mtGBLUP model, the phenotypes from environment 1 (Env 1) were treated the same as before, and the phenotypes from environment 2 (Env 2) were treated as training set 2, described in Figure 1. The variance components were estimated by the training set, which was used to calculate the genetic estimated breeding values (GEBVs) of the test set. The prediction accuracy of these strategies was evaluated by the Pearson’s Correlation of the GEBVs and the observed phenotypes of test set. The CV was replicated 20 times. Finally, the average prediction accuracy for each approach was utilized to evaluate the predictive ability.

## 3. Results

### 3.1. Statistical Summary and the Estimated Genetic Parameters for All Traits

The descriptive statistics and the estimated genetic parameters for all agronomic traits for DS and WS are shown in Table 1. The result shows that the mean of phenotypes is different between DS and WS for the same trait. Besides, these traits differed in their narrow-sense heritability. For example, the narrow-sense heritability of PH and FLW are high, and Exs and PnN are low. However, the narrow-sense heritability is differential for the same trait between DS and WS (i.e., the narrow-sense heritability of PH is 0.46 ± 0.09 and 0.39 ± 0.08 for DS and WS, respectively). In addition, a great number of traits possess high genetic correlation (~1) between DS and WS. Furthermore, there is a median/low phenotypic correlation for all traits between DS and WS, such as Exs and PnN.

### 3.2. Prediction Accuracy for Three Genomic Preidcition Models

**Figure 2:**
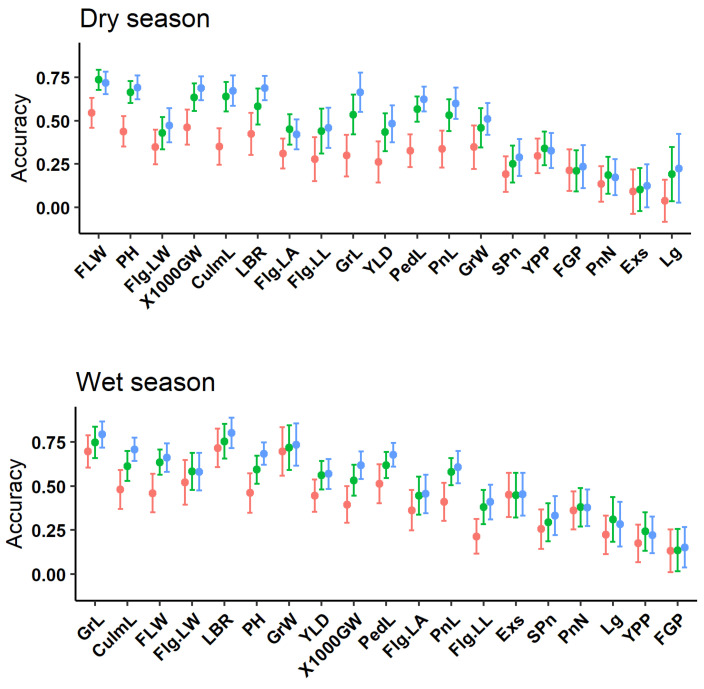
The predictive accuracy of three genomic prediction models for DS and WS (sorted by the narrow-sense heritability). The labels of *x*-axis denote 19 traits’ names. The *y*-axis represents the predictive accuracy for three models. Each point represents the mean prediction accuracy of 100 times (n = 5 × 20 times). The error bar indicates the standard deviation of prediction accuracy. The dot color of red, green, and blue represent GBLUP, GFBLUP, and mtGBLUP model, respectively.

The prediction accuracy among GBLUP, GFBLUP, and mtGBLUP is displayed in Table S1 and Figure 2. The percentage of top markers with best prediction accuracy of GFBLUP model is reported. The result shows that the prediction accuracy of mtGBLUP model is better than GBLUP model for all traits (Figure 2). In addition, it is similar to the GFBLUP model for the majority of traits (30 out of 38). Furthermore, the mean of prediction accuracy of mtGBLUP model for all traits is greater than the GBLUP (+14.53%) and GFBLUP (+3.23%) model (Figure S3). The result shows that the prediction accuracy of mtGBLUP model shows advantages to the other models. Compared with the GBLUP model, the prediction accuracy of GFBLUP model and mtGBLUP model is preferable for the majority of traits. In addition, the prediction accuracy of GFBLUP model is slightly similar to mtGBLUP model (Figure S3, the median of deviation of prediction accuracy between mtGBLUP and GFBLUP model is +0.0342 and +0.0248 for dry and wet season, respectively). Furthermore, the prediction accuracy is different, even for the same trait at different environments.

We found that the trend of prediction accuracy for GBLUP model is different between the markers selected specifically or randomly, which is shown in Figure S2. For the majority of traits, the prediction accuracy of GBLUP model with markers selected specifically shows a trend of first rising and then decreasing, while a GBLUP model with markers selected randomly implies a tendency of first increasing and then maintaining stability.

### 3.3. The Relationship between the Prediction Accuracy and the Genetic Parameters

**Figure 3:**
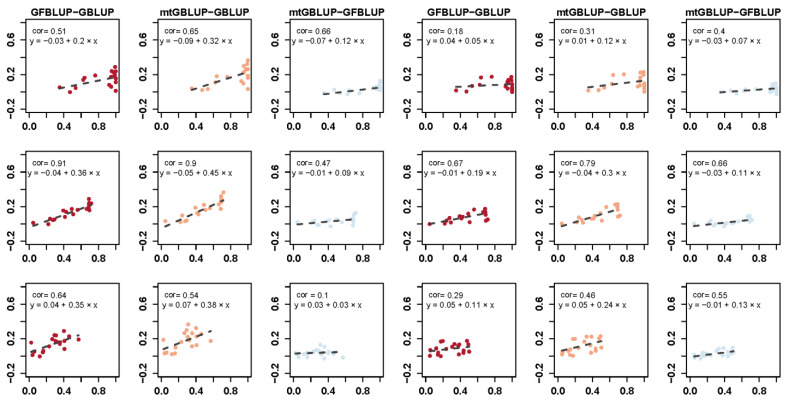
The relationship between the predictive accuracy and estimated genetic parameters for the different models for DS (three columns at left) and WS (three columns at right). *y*-axis is the deviation of predictive accuracy between models. The correlation coefficient is the Pearson correlation calculated by the prediction accuracy and estimated genetic parameter. Each point represents the prediction accuracy of mean for each trait and the dash line indicates the linear regression fitness between the prediction accuracy and estimated genetic parameter.

Figure 3 displays the correlation between the genetic parameters and the deviation of the prediction accuracy between different models. First of all, the fitness between the deviation of the prediction accuracy and the estimated genetic parameters has a similar trend between DS and WS. Secondly, the prediction accuracy of mtGBLUP model and GFBLUP model is strongly preferable to GBLUP model. In addition, the prediction accuracy of mtGBLUP model is slightly similar to the GFBLUP model. Finally, compared with other parameters, the deviation of both the GFBLUP model and mtGBLUP model with the GBLUP model is more associated with the phenotypic correlation.

## 4. Discussion

The objective of this study was to compare the performance of different GP models and assess the utilization strategies of GP models in different scenarios. Compared with other models, the mtGBLUP model demonstrated the best performance for the majority of traits. On the other hand, the optimal GP model could be selected according to different availability of multiple environment phenotypes.

Compared with other models, the mtGBLUP model presents a better prediction accuracy for all traits. One of the reasons for improving prediction accuracy is that joint prediction of multiple traits could benefit from the high genetic correlation between the traits, which is consistent with the previous studies [19,28]. In addition, mtGBLUP model makes full use of the multiple environment phenotypes, which could greatly improve the prediction accuracy. However, the prediction accuracy of mtGBLUP model is not only influenced by the genetic correlation but affected by the availability of multiple environment phenotypes. In summary, the prediction performance of mtGBLUP model is generally better than the other genomic prediction models on the basic of the complete availability of multiple environment phenotypes.

Due to partial availability or unavailability of multiple environment phenotypes, mtGBLUP model is not applicable. With the purpose of improving prediction accuracy, the additional information, such as the GWAS results, originated from the other environment, could be utilized. In this study, the result shows that integrating the GWAS results originated from the other environment (GFBLUP model) could strongly increase the prediction accuracy comparing to GBLUP model. Besides, the prediction accuracy of GFBLUP model is slightly close to mtGBLUP model (Figure 2 and Table S1). It indicates that although other environment phenotypes could not be combined straightly, the information from other environments, such as the GWAS results, could be integrated to improve the prediction accuracy. Therefore, when the multiple environment phenotypes are partially unavailable, the GFBLUP model could slightly substitute the mtGBLUP model.

The prediction accuracy would be improved by considering the genetic architecture of complex quantitative traits. Zhang et al. [11] suggested that integrating public GWAS results into the standard GBLUP model could improve the prediction accuracy. Several studies showed that considering the genomic features could optimize the prediction accuracy, such as gene ontology [14] and gene annotation [15]. In our study, it showed that incorporating GWAS results from other environments into GBLUP model could increase the prediction accuracy, which was consistent with the recent study [29]. Besides, Teng et al. [30] showed that optimizing the casual gene to the GP model could improve the prediction accuracy. Furthermore, comparing to GBLUP model with markers selected randomly, GBLUP model with markers selected specifically could increase the prediction accuracy for some traits, which demonstrated that the casual variants might played a more significant role in GP. In summary, aiming to improve the prediction accuracy, it is important to consider the genetic architecture of complex quantitative traits.

With the increase of the narrow-sense heritability of the traits, the prediction accuracy is synchronously improved (Figure 2), which is in line with the previous study [31]. In addition, a small number of traits display the poor prediction accuracy for all models, which might be caused by the distribution of the phenotypes. Besides, compared with other genetic parameters, the deviation of prediction accuracy between GFBLUP model and GBLUP model is more correlated to the phenotypic correlation (Pearson’s correlation is equal to 0.91 and 0.67 for DS and WS, respectively.) (Figure 3). One of the critical reasons is that the GWAS information takes full advantage of the phenotypes from other environments.

The GWAS results are shown in Figure S1. For the majority of traits, with the increase of the marker density, the prediction accuracy of GBLUP model whose marker selected randomly first increased and then stabilized (Figure S2) was consistent with the previous study [32]. In addition, the prediction accuracy of the GBLUP model, whose selected marker specifically first raised and then decreased, implies that an excess of marker density might interfere with the prediction accuracy of GP. In summary, the prediction accuracy of GBLUP model is not only influenced by the marker density, but also largely affected by the casual variants.

## 5. Conclusions

In summary, genomic prediction models considering multiple environment phenotypes (GFBLUP and mtGBLUP model) demonstrated better prediction accuracy. We could utilize different genomic prediction strategies according to availability, the GWAS summary statistics, or phenotypes from multiple environment phenotypes.

## Figures and Tables

**Table 1:** Descriptive statistics and estimated genetic parameters for all measured traits.

Trait	Dry Season			Wet Season			C_g_ ± s. e.	C_p_
	N	Mean	$h_{d}^{2}\pm s.e.$	N	Mean	$h_{w}^{2}\pm s.e.$		
PH	342	105.6	0.46 ± 0.09	370	119.7	0.39 ± 0.08	0.973 ± 0.03	0.691
FLW	342	84.17	0.57 ± 0.09	370	92.39	0.48 ± 0.09	0.763 ± 0.07	0.687
Lg	342	1.274	0.02 ± 0.04	370	1.588	0.12 ± 0.06	1.000 ± 0.65	0.393
Exs	342	1.22	0.04 ± 0.03	370	1.447	0.17 ± 0.06	1.000 ± 0.45	0.046
CulmL	342	82.47	0.40 ± 0.09	363	95.62	0.49 ± 0.09	0.993 ± 0.02	0.690
PnN	342	12.73	0.06 ± 0.05	370	12.58	0.14 ± 0.06	0.352 ± 0.37	0.291
PedL	342	3.204	0.29 ± 0.08	370	4.356	0.31 ± 0.07	0.953 ± 0.05	0.693
PnL	342	23.47	0.27 ± 0.08	370	24.14	0.20 ± 0.07	0.990 ± 0.05	0.688
Flg LL	342	29.34	0.32 ± 0.09	370	31.65	0.18 ± 0.07	0.640 ± 0.17	0.560
Flg LW	342	1.146	0.41 ± 0.09	370	1.155	0.44 ± 0.08	0.925 ± 0.07	0.413
Flg LA	342	22.65	0.35 ± 0.10	370	24.54	0.24 ± 0.08	0.624 ± 0.16	0.427
SPn	342	77.01	0.16 ± 0.07	370	107.1	0.14 ± 0.06	0.948 ± 0.17	0.241
FGP	342	977.6	0.12 ± 0.06	370	1065.5	0.05±0.04	0.472 ± 0.39	0.220
GrL	342	9.693	0.31±0.08	370	9.794	0.50 ± 0.08	1.000 ± 0.03	0.718
GrW	342	2.301	0.23 ± 0.07	370	2.436	0.38 ± 0.08	1.000 ± 0.05	0.509
LBR	342	4.253	0.36 ± 0.08	370	4.081	0.44 ± 0.08	1.000 ± 0.04	0.696
1000 GW	342	24.42	0.41 ± 0.09	370	24.98	0.33 ± 0.08	0.955 ± 0.04	0.665
YPP	342	24.15	0.16 ± 0.06	370	29.01	0.05 ± 0.04	0.536 ± 0.33	0.275
YLD	342	4917	0.30 ± 0.09	370	4720	0.38 ± 0.09	0.946 ± 0.06	0.492

Note: $h_{d}^{2}$ and $h_{w}^{2}$ is the narrow-sense heritability of dry season and wet season; s.e. is the standard error of the narrow-sense heritability; $C_{g}$ and $C_{p}$ are the genetic and phenotypic correlation between dry season and wet season for each trait. Plant height (PH), flowering date (FLW), culm length (culmL), number of effective tiller or panicle per plant (PN), panicle length (PL), flag leaf length (FlgLL), flag leaf width (FlgLW), flag leaf area (FlgLA), number of spikelets per panicle (SPn), number of filled grain per plant (FGP), grain length (GrL), grain width (GrW), grain length-breadth ration (LBR), lodging score (LG), peduncle length (PedL), panicle exertion rate (Exs), 1000 grain weight (1000 GW), yield per plant (YPP), and grain yield per plot (YLD).

## Data Availability

Publicly available datasets were analyzed in this study. This data can be found here: http://www.ricediversity.org/data/index.cfm, accessed on 30 December 2019.

## Supplementary Materials

The following supporting information can be downloaded at https://www.mdpi.com/article/10.3390/genes13050722/s1, Figure S1: The Manhattan plot of the GWAS results for all traits. Figure S2: The trend of predictive accuracy by the marker density selected randomly or specifically for GBLUP model for dry season and wet season. Figure S3: The deviation of prediction accuracy for three genomic prediction models. Table S1: The predictive accuracy of three models for all traits for dry season and wet season.
