# Supplementary material for: Utilization Strategies of Two Environment Phenotypes in Genomic Prediction
Source: Genes (Basel). 2022 Apr 20;13(5):722. doi: 10.3390/genes13050722 (PMC9141986; doi:10.3390/genes13050722)
Supplement: Supplementary file 1 [file genes-13-00722-s001.zip › Supplemental materials information.pdf]

Supplemental materials

Supplemental file 1: Table S1

Format: .csv

Title: The predictive accuracy of three models for all traits for two seasons

Description: The predictive accuracy of GBLUP, GFBLUP, mtGBLUP model for all traits for dry and wet season (Mean ± S.E.).

Supplemental file 2: Table S2

Format: .csv

Title: The marker numbers which selected from 1% to 60% by randomly or specifically.

Description: The markers selected by randomly or specifically were utilized to construct genomic relationship matrices G_F and G_R and to evaluate the genomic prediction accuracy.

Supplemental file 2: Figure S1

Format: .tiff

Title: The Manhattan plot of the GWAS results for all traits

Description: The Manhattan plot of the GWAS results for all traits for dry season (left) and wet season (right).

Supplemental file 3: Figure S2-DS and Figure S2-WS

Format: .tiff

Title: The trend of predictive accuracy by the marker density selected randomly or specifically for GBLUP model

Description: The trend of predictive accuracy by the marker density selected randomly (blue line) or specifically (red line) for GBLUP model, respectively. y-axis is the predictive accuracy. The solid line, dashed line and dotted line indicate the prediction accuracy of GBLUP, GFBLUP and mtGBLUP, respectively. The solid line, dashed line and dotted line indicate the prediction accuracy of GBLUP, GFBLUP and mtGBLUP,

respectively.

Supplemental file 4: Figure S3

Format: .tiff

Title: The deviation of prediction accuracy for three genomic prediction models.

Description: Each red point represents one trait from dry or wet season.
