# Supplementary figures and images for: Utilization Strategies of Two Environment Phenotypes in Genomic Prediction
Source: Genes (Basel). 2022 Apr 20;13(5):722. doi: 10.3390/genes13050722 (PMC9141986; doi:10.3390/genes13050722)

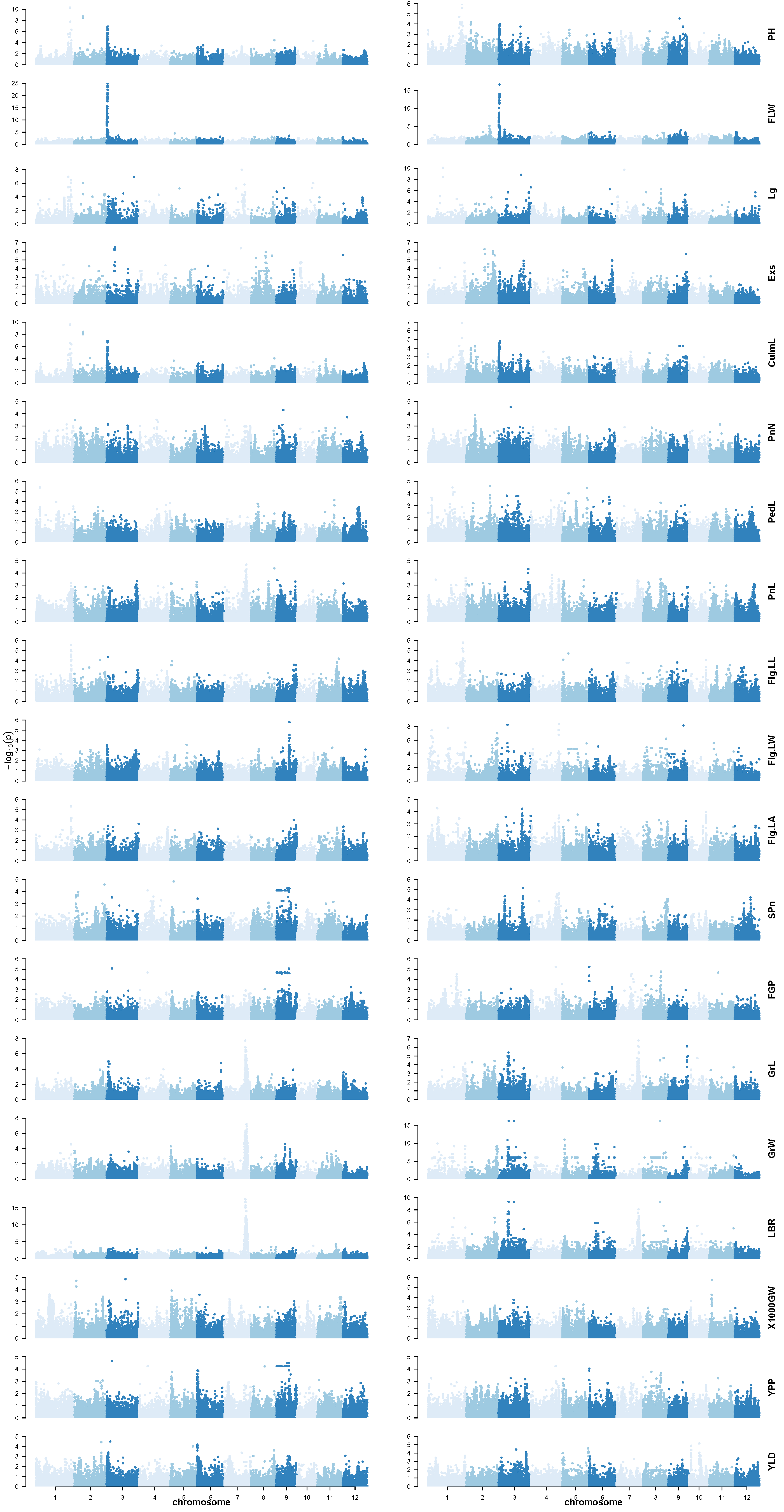

Supplement: Supplementary file 1 [file genes-13-00722-s001.zip › Figure S1.tiff]

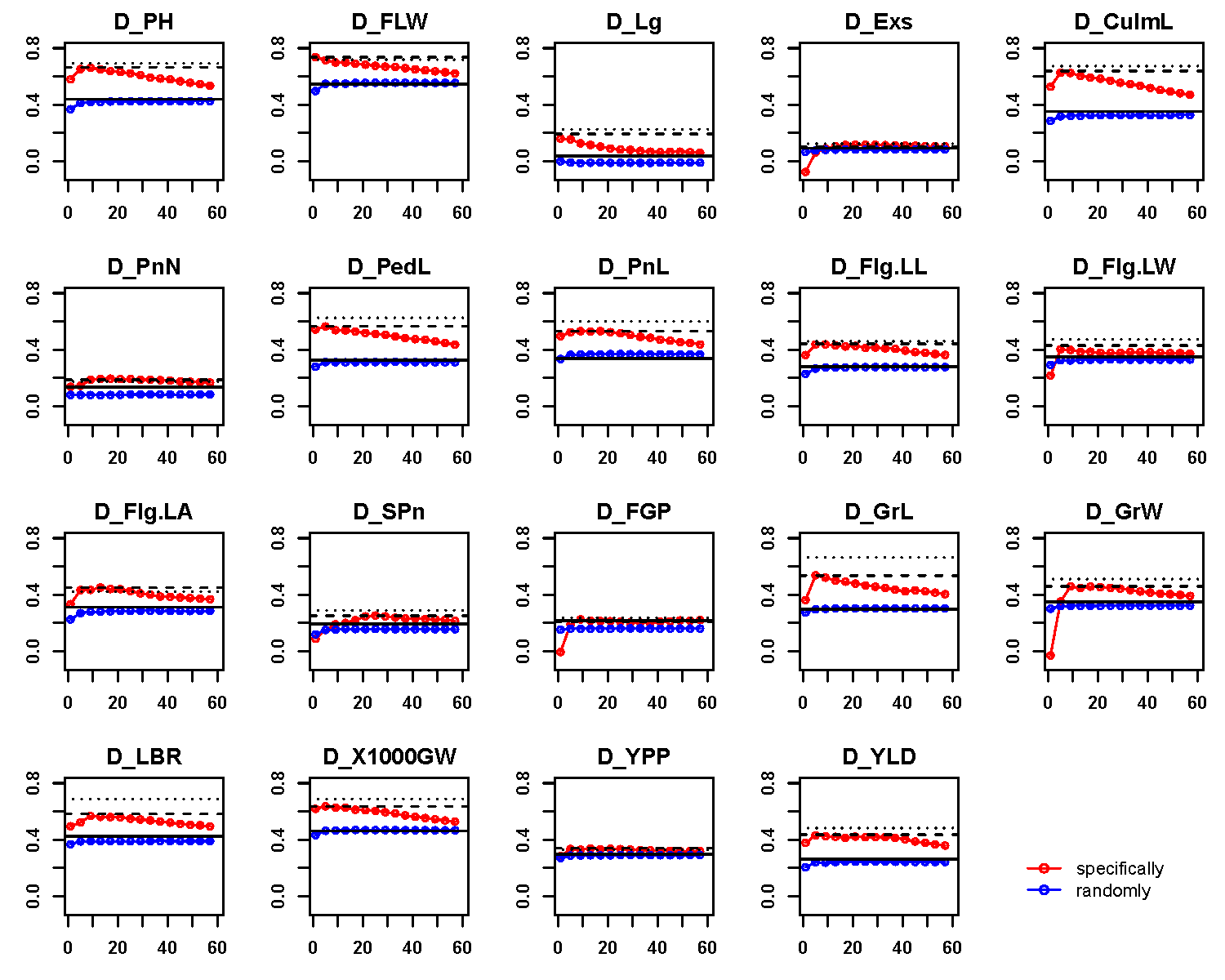

Supplement: Supplementary file 1 [file genes-13-00722-s001.zip › Figure S2-DS.tiff]

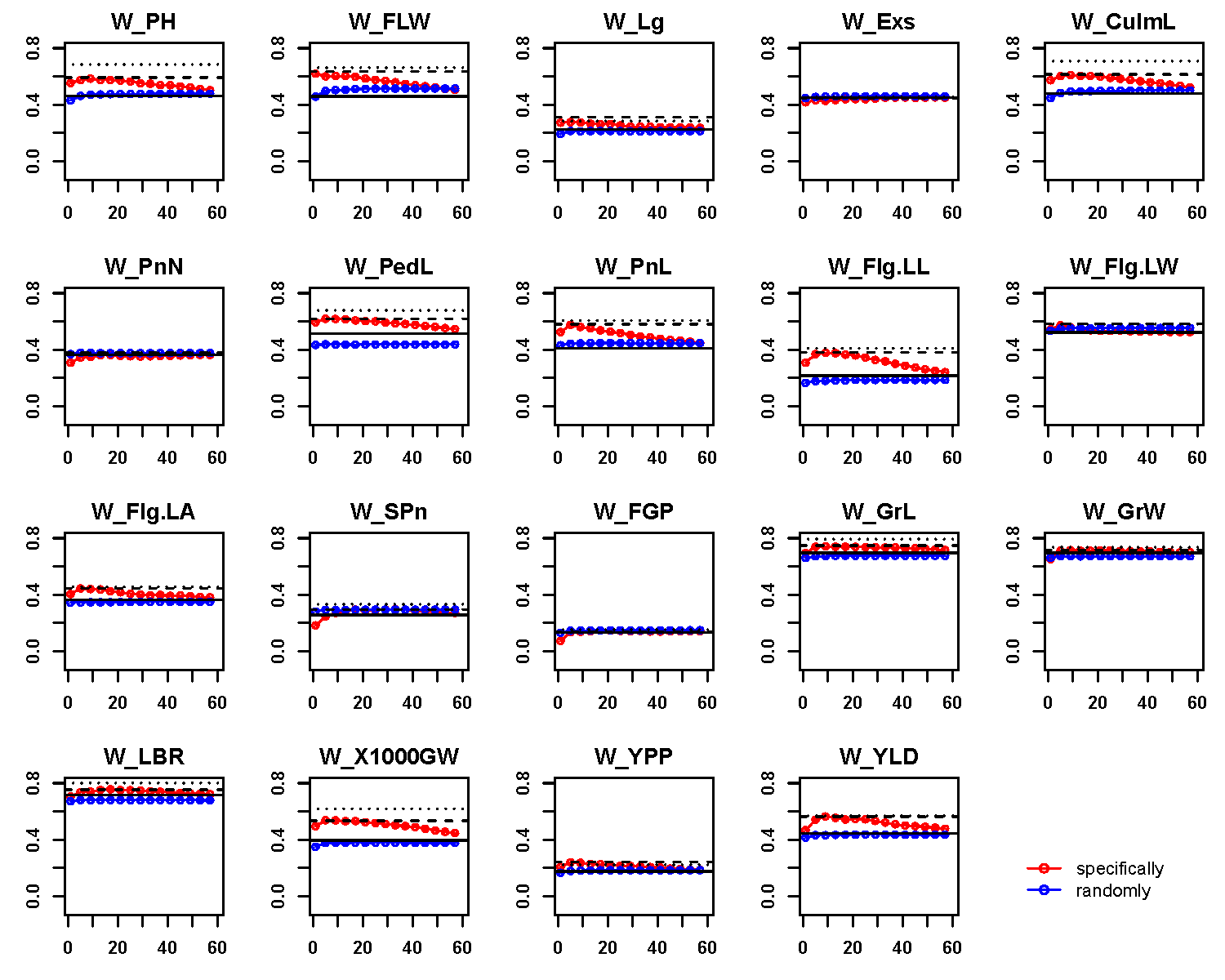

Supplement: Supplementary file 1 [file genes-13-00722-s001.zip › Figure S2-WS.tif]

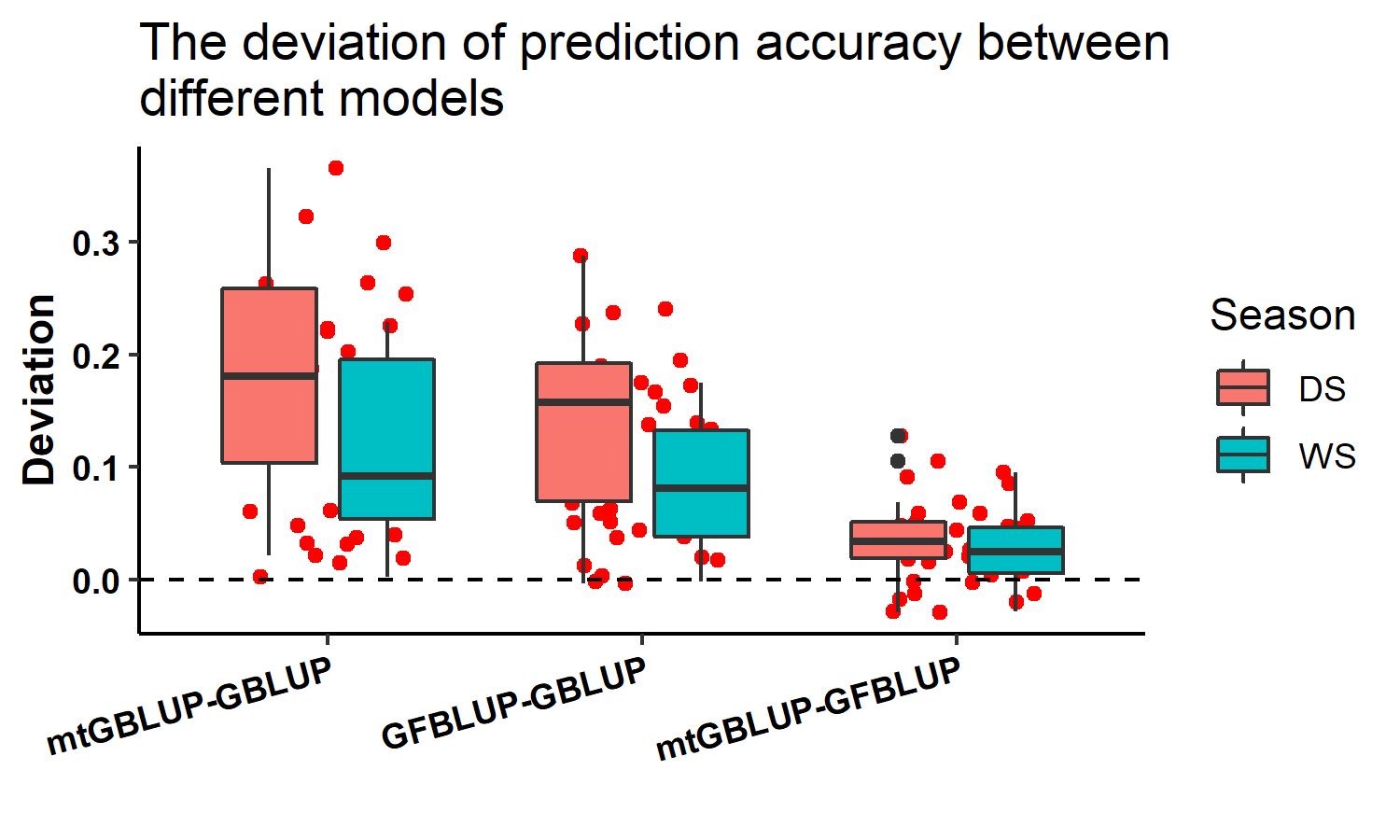

Supplement: Supplementary file 1 [file genes-13-00722-s001.zip › Figure S3.tiff]
